# Experience affects novel object aversion and the use of human cues in rural and urban American crows

**DOI:** 10.1093/beheco/arag135

**Published:** 2026-10-22

**Authors:** Dave J Colucci, Anne B Clark

**Affiliations:** Department of Biological Sciences, Binghamton University, 4400 Vestal Parkway East, PO Box 6000, Binghamton, NY 13902, United States; Department of Biological Sciences, Binghamton University, 4400 Vestal Parkway East, PO Box 6000, Binghamton, NY 13902, United States

**Keywords:** neophobia, flight initiation distance, urban, corvid, gaze, novelty

## Abstract

Population differences in behavior can result from experience, which can be drastically different between environments such as urban and rural areas. Urban areas may be more complex, contain more human-made objects but fewer natural objects, or have more nonthreatening people. Consequently, animals in urban and rural populations may differ in neophobia levels, what is considered novel, and attention to human behavior. We conducted 2 experiments with a common urban bird, the American crow (*Corvus brachyrhynchos*). On the home territories of urban and rural crow family groups, we presented objects that were either equally novel in both environments or more common in one environment than the other. Separately, we measured flight initiation distance (FID) from an approaching person using either a direct or averted, less threatening gaze for individual crows across an urban gradient. We found that urban crows were more neophobic than rural crows, as measured by the probability of feeding near an object that is equally novel in both environments. There were no differences in approach and feeding latencies for the least neophobic family member. Both urban and rural crows were less averse to objects that were more common in their respective environments. In the FID experiment, crows in highly urbanized areas had FIDs 25 m shorter for an indirect gaze, while rural crow FIDs were unaffected by gaze. FIDs were best predicted by crows' experiences with people in a relatively large area around each trial. These results highlight how experience must be accounted for when considering population differences in behavior.

## Teaser

We tested whether urban and rural American crows differ in two behaviors affected by experience: feeding near unusual objects and fleeing from an approaching person. We show that urban crows are less likely than rural individuals to feed near a novel object, while crows in both environments avoid objects that they are less familiar with. Separately, we show that only urban crows use the direction of an approaching person’s gaze when deciding when to escape.

## Introduction

Intraspecific behavioral differences are often driven by environmental variation, and such differences can reveal how organisms interact with the external world and respond to changes in it. The contrast between natural and urban areas is a major source of such environmental variation (Oke 1973; McKinney 2002, 2006; Grimm et al. 2008; Haddad et al. 2015). Because both urban land area and the human population are expected to grow rapidly over the next several decades (United Nations Department of Economic and Social Affairs Population Division (ST/ESA/SER.A/420) 2019; Chen et al. 2020), it is important to understand how organisms can adjust their behavior to these changes. Behavioral changes are often an organism's first response to environmental change (Tuomainen and Candolin 2011; Wong and Candolin 2015) and can influence the potential for subsequent evolutionary adaptation (Botero 2026).

Approximately 20% of bird species occur in urban areas (Aronson et al. 2014) and, for many of these, city life is accompanied by behavioral changes. While there is evidence that some of these shifts have evolved (Lambert et al. 2021), many likely reflect preexisting norms of reaction and especially the different experiences that individuals accumulate while living in urban or rural environments. Experience affects many types of behaviors, and urban behavioral shifts may help organisms persist in this human-created environment.

One behavior that is shaped by experience is object neophobia, an aversive reaction to a novel object, usually measured as the decreased power of an attractive stimulus when such an object is present (Greenberg and Mettke-Hofmann 2001). An object's novelty depends on an individual having no prior experience with it, but an individual's broader experiences can also affect its level of neophobia. For instance, individual animals that are experimentally exposed to a series of novel objects, live in constantly changing environments, or are exposed to more stimuli show less avoidance of novel objects (see review in Corey 1978). These conditions are often used to describe urban areas, suggesting that urban individuals will have reduced neophobia simply due to the background of change and object turnover in their environment. Neophobia varies among species (Miller et al. 2025) and may play an adaptive role in matching populations to their environments. For instance, Mettke-Hofmann et al. (2013), in a study of 6 blackbird (Icteridae) species, found that both nonmigratory species and nonmigratory populations within species tend to be less neophobic than migratory ones (Mettke-Hofmann et al. 2013). The authors suggested that year-round residents have more to gain by exploiting novel resources, while, for migrants, any benefit is short-term and outweighed by exposure to novel dangers. Similar cost-benefit logic is often used to argue that less neophobic individuals will be more successful in cities, as they will be able to exploit novel, human-sourced food resources (Sol et al. 2011).

Experiments testing these predictions within different species have found decreased, unchanged, or even increased object neophobia in urban populations (see reviews in Greggor et al. 2015; Griffin et al. 2017). These discrepant findings could relate to differences in which parts of the external world are relevant to each species. Urban areas may, for example, be more complex for some species, but possibly simpler for others. Similarly, the costs and benefits of neophobia might change differently in urban areas for different species, causing neophobia levels to shift in different directions. Experimental methodology can also affect comparisons of neophobia, as some approaches accidentally measure traits other than neophobia (Réale et al. 2007; Greggor et al. 2015).

An overlooked methodological concern when object neophobia is measured in populations from 2 environments is how to account for differences in the types of objects experienced in each, as these differences make it difficult to choose an object that is equally novel to both populations. Using artificial objects made from human-produced materials has been suggested as a reasonable way to ensure their novelty (Greenberg and Mettke-Hofmann 2001), and this approach has been used to compare neophobia in urban and rural populations (Audet et al. 2016; Greggor et al. 2016a, ; Ducatez et al. 2017). However, since artificial objects are more common in urban areas, urban individuals are likely to have more experience with their distinctive properties, such as bright colors, shiny or smooth surfaces, and sharp angles. If urban individuals have more experience with these artificial properties, any object possessing them may be less novel to urban individuals than to rural individuals. To measure differences in neophobia between these populations, care should be taken to choose an object that does not have the distinct properties of artificial objects; otherwise, population differences in aversion may reflect differences in experience, not neophobia. The consequence of differences in experience between 2 populations should be clear if a series of objects are presented, some of which are more novel for one population than the other. Animals respond with increasing aversion to increasing novelty (Corey 1978), so a series of objects ranging from familiar to novel from the perspective of urban individuals could be ranked in a different order of novelty for rural individuals, with corresponding differences in aversion.

In addition to neophobia, experience also affects the distance at which a bird will flee an approaching person, primarily by altering the relative costs of fleeing, such as lost time foraging, and the benefits of reducing predation risk (Ydenberg and Dill 1986; Frid and Dill 2002). In urban areas, birds have more frequent and more positive experiences with humans (Clucas and Marzluff 2012) and typically allow closer approaches, as measured by an individual's flight initiation distance (FID) (Lowry et al. 2013; Samia et al. 2015). Birds may evaluate risk by observing the behavior of an approaching person and can have longer FIDs when the approaching person gazes directly at the focal bird instead of gazing away (Bateman and Fleming 2011; Clucas et al. 2013), as a predator that fixes its gaze on a subject is likely to pose a greater threat (Davidson et al. 2014; Davidson and Clayton 2016). Use of human gaze increases with experience with humans in chimpanzees (Call et al. 2000) and Bobwhite quail (*Colinus virginianus*) (Jaime et al. 2009), so urban individuals may be more likely to use the gaze of an approaching person to inform their escape decisions. This has rarely been tested (but see Goumas et al. 2020). Comparisons of urban and rural FIDs also require experimenters to use proxies for experience with humans, often human population density or urban land-use (Rodriguez-Prieto et al. 2009; Mikula 2014; Carlen et al. 2021). However, the size of the area around an FID trial in which a proxy is measured is often not justified (but see Carlen et al. 2021) and is likely species-specific. Flocking also affects predation risk and may reduce FID if individual risk is diluted in a group, or increase FID if groups detect predators earlier in their approach (Ydenberg and Dill 1986). Unsurprisingly, different effects of flock size have been found for different species (Morelli et al. 2019; Ardila-Villamizar et al. 2022).

American crows (*Corvus brachyrhynchos*) established successful urban breeding populations about 70 yrs ago (Johnston 1961; Marzluff et al. 2001; McGowan 2001), and urban crows are often observed manipulating human refuse and incorporating anthropogenic foods into their diets (Marzluff et al. 2001; Withey and Marzluff 2005; Campbell 2008). During the summer, crows in our study population in Ithaca, NY, live in cooperatively breeding groups (McGowan 2001; Clark et al. 2006; Townsend et al. 2009), as do crows throughout eastern North America (Kilham 1989; Chamberlain-Auger et al. 1990). In Ithaca, crow territories are on average equivalent in area to circles with diameters of 0.31 and 0.67 km in suburban and rural areas, respectively (McGowan 2001). However, crows' experiences extend beyond their territories, as they are highly mobile. They will join conspecifics on neighboring territories to mob predators, and, during the nonbreeding season, will often move long distances and between habitats to migrate, join foraging aggregations, or join night roosts (Townsend et al. 2018).

American crows are members of the family Corvidae, a group known to be curious (Heinrich 1995; Miller et al. 2015; Lambert et al. 2017) and intelligent (Emery and Clayton 2004; Emery 2006; Kabadayi et al. 2017); they are also known for being very neophobic (Greenberg and Mettke-Hofmann 2001). For example, a study in the United Kingdom found that corvid species as a group visited a feeding table less frequently and fed less quickly than noncorvid species when a novel object was present (Greggor et al. 2016a, ). Urban and rural birds in this study did not differ in their responses to the novel object. Similarly, the degree of urbanization did not affect the latency for the first American crow in a group to approach an anthropogenic food and artificial object (Merz et al. 2025). In contrast, captive individuals of corvid species that use urban areas have lower neophobia than captive individuals of those species that do not (Miller et al. 2022), suggesting that lower neophobia may be especially important for corvids' ability to take advantage of novel resources. This prediction has rarely been tested by comparing urban and rural populations of a single corvid species.

Historically, humans persecuted American crows as agricultural pests (Marzluff and Angell 2005), and in rural areas, they are still often discouraged from visiting homes (Clucas and Marzluff 2012) and are hunted for sport. Crows and other corvids in rural areas have much longer FIDs than in urban areas and have the longest FIDs where they are most persecuted (Kenney and Knight 1992; Clucas and Marzluff 2012; Fujioka 2020). In Seattle, WA, urban crows respond to a direct human gaze with shorter FIDs than to an indirect gaze (Clucas et al. 2013), but it is unclear if rural American crows use gaze direction in FID decisions. Flock size did not affect FID in experiments with several corvid species (Clucas et al. 2013; Fujioka 2020; Novcić 2023), but Randler (2008) found that both total flock size and conspecific flock size affected FID in the carrion crow (*Corvus corone*)—hooded crow (*Corvus cornix*) species complex. Time of year affects FID in several corvid species (Poddubnaya et al. 2019), but its effect on the FIDs of American crows has not been studied.

Here, we asked 2 main questions about neophobia: (i) Do urban crows have lower neophobia levels than rural crows? (ii) Are the responses of both urban and rural crows contingent on the different experiences we expect crows to have in each environment? We then asked 2 related sets of questions about crow FIDs: (i) Do rural crows, like urban crows, allow an experimenter to approach more closely when using an indirect rather than a direct gaze? (ii) For both urban and rural crows, at what spatial scale do experiences with humans most strongly affect FID, and do flock size or time of year affect FID? Thus, we are interested in both the differences in behavior between urban and rural crows and how experience affects those behaviors.

## Methods

### Object neophobia

#### Object choice

To ensure that crows would react to the novelty of the object and not any other characteristic, we chose objects that are roughly the same size and level of complexity (Berlyne 1950; Greenberg 1983), and none that contain potentially aposematic colors or patterns that might resemble eyes (Carter et al. 2008; Hausberger et al. 2018). In Fig. 1, we show the 4 objects that were used, arranged along a scale from familiar to novel for each environment. We used a rock to test the effect of a familiar object. At the high end of our novelty scale, we used a faux-wood lamp base. It was chosen because it lacks common properties of artificial objects such as bright colors, shiny surfaces, or sharp angles. In the 2 intermediate positions on our novelty scale, we used a piece of corn-in-husk and a paperboard take-out box. We expected the corn-in-husk to be more novel to urban crows, while the take-out box would be more novel to our rural crows. Thus, the lamp served to test our hypothesis that urban crows had lower neophobia than rural crows, while the corn and box tested our expectation that crows' responses would be dependent on our assumptions about their experiences with different types of objects.

**Figure 1:**
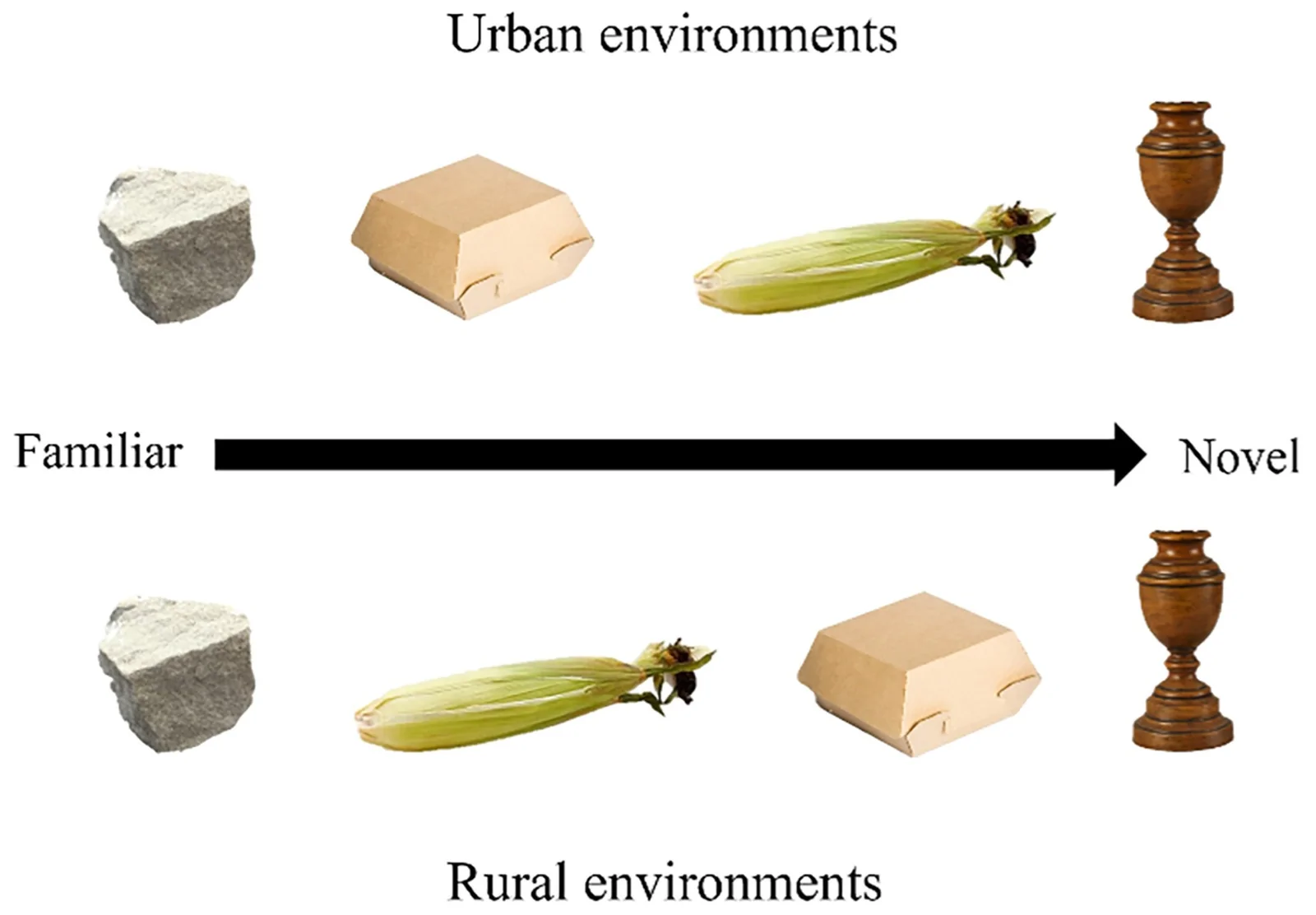
Objects used in the neophobia experiment in order of their expected novelty in each environment. From left to right in rural environments: a rock, a piece of corn-in-husk, a paperboard take-out box, and a lamp base.

After trials were complete, we assessed the presence and turnover of potentially novel objects at one randomly selected site in each environment. At each site, we surveyed both sides of a 1.61 km road route once per week for 3 wks, recording each artificial (ie human-made) object and whether it was present during each weekly survey.

#### Fieldwork

We established feeding sites on the territories of 10 urban and 10 rural crow families in and around the city of Ithaca, NY, in 2019 and 2020. Rural sites were on plots of private or public land in the agricultural or natural areas in the Town of Dryden. Urban sites were in public parking lots, dead end streets, and parks in residential and commercial areas in the Town of Ithaca, the Village of Lansing, or the Village of Cayuga Heights. Measurements of the percent urban land cover and the human population density around each site are provided in Table S1 (see below for GIS methods). For simplicity, we refer to these sites as urban and rural but acknowledge our urban areas are more like suburban than downtown urban areas (Marzluff et al. 2001; McGowan 2001). Sites were chosen that were relatively free from human traffic, predator activity (red foxes, *Vulpes vulpes*; red-tailed hawks, *Buteo jamaicensis*), and competition (gray squirrels, *Sciurus carolinensis*; various *Larus* gulls).

Each family's feeding site was baited with 2 cups of raw, unsalted peanuts in the shell, which American crows will eat or cache, spread out in a circle with a radius of 0.5 m. Sites were baited 4 to 6 days per week until crows fed on 3 consecutive attempts. We then conducted 3 video-recorded baseline trials with no object present (bait only) and 4 object trials (bait and object). We conducted only 1 trial per day with each family. Each trial began after 6 AM and no later than 8 AM. During object trials, the object was placed in the center of the bait circle. Object presentation order was pseudo-randomized, with the restriction that each object appeared at least once in each chronological position in each environment. Video recordings were made using a Canon VIXIA HF R600 camcorder, placed at least 3 m from the center of the bait site and mounted on a tripod or existing structure (telephone poles, fence posts, etc.). We attempted to hide the camcorder by setting it back into the vegetation, wrapping it with camouflage tape, or affixing vegetation. Each trial was observed by the experimenter (D.J.C.) from a minimum distance of 50 m. Trials ended when the bait was exhausted and the experimenter observed the crows leaving the area, or when the battery on the camcorder ran out (max trial length of ∼1.5 h). All trials were done in July or August, after the breeding season concluded but while families could include mobile, dependent fledglings. All trials for a family were completed within the same calendar year.

Three measures were taken in each trial. First, we recorded the latency from when the experimenter first saw crows near the site to when the first crow in a family approached within 3 m of the object (or the center of the bait circle in trials with no object). Next, we recorded the latency from when the first crow approached within 3 m of the bait/object until the first time that a crow in the family fed. Last, the experimenter used their direct observations and the video recordings to determine the minimum number of crows that came within 3 m of the object and the minimum number that successfully fed. Feeding was defined as successfully picking up a peanut, as crows frequently left the area within 3 m of the bait/object with whole peanuts to cache or consume them elsewhere.

#### Statistical analyses

Statistical analyses were carried out using R 4.4.1 (R Core Team 2024). The latency for the first crow in a family to approach within 3 m of the bait/object and the latency for the first crow in a family to feed after a family member approaches within 3 m of the bait/object were both analyzed using Cox proportional hazards mixed-effects models from the package *coxme* (Therneau 2024) with crow Family as a random effect. Fixed effects were Environment (urban or rural), Object (none, rock, corn, box, and lamp), and their interaction. We analyzed the proportion of crows that fed with a GLMM with a binomial error distribution and logit link using the package *lme4* (Bates et al. 2015) with the same fixed and random effects as above. The model failed to converge due to complete separation in the data (all crows fed in urban × rock). To handle complete separation, we fit a Bayesian binomial mixed-effects model using the package *brms* (Bürkner 2017). Population-level (fixed) effects were assigned normal priors with a mean of 0 and a standard deviation of 2.5. To inform the prior on the group-level (random) effect of Family, we fit the binomial GLMM using *lme4* after excluding rock trials to eliminate complete separation. That model indicated that variance among families was low, so we fit the Bayesian model using a moderately informative prior for Family, specified as a Student’s *t*-distribution with 3 degrees of freedom, a mean of 0, and a standard deviation of 1. For each of the models above, we conducted contrasts within each Environment of adjacent pairs of objects along our expected novelty scale (Fig. 1), and of the 2 levels of Environment for each level of Object. We also evaluated whether urban and rural crows reacted differently to the box and corn by testing for an interaction between Environment and those 2 levels of Object. All contrasts were performed with the *emmeans* package (Lenth 2025).

Differences in the turnover of litter items from the litter survey were determined by summing the number of times each object was observed across the 3 survey weeks and comparing the mean number of observations between the urban and rural transects using a 2-sample *t*-test.

The percentage of urban land cover and human population density around each site were determined within a 245.5 m buffer, corresponding to the average radius of 2 circles with areas equivalent to those of rural and urban crow territories in our study area (McGowan 2001). Measurements were made in QGIS (QGIS Development Team 2023) using data from the North American Land Change Monitoring System (Commission for Environmental Cooperation (CEC) 2023) and the 2020 United States Census (United States Census Bureau 2020). Population density around a site was the sum of population densities for census blocks in the buffer, each weighted by the proportion of the buffer that they occupied.

### Influences on FID

#### Fieldwork

Trials were carried out in urban and rural areas in upstate New York, United States, and in Quebec, CA. We only collected data in June, July, and August. This is the period after breeding, but when crows are typically on their home territories. We aimed to avoid retesting the same individuals by conducting trials at least 500 m apart, except when testing banded individuals in our long-term study population in Ithaca, NY (McGowan 2001; Clark et al. 2006). Trials were done in 2019, 2022, and 2023, but not during the COVID lockdown in 2020 or in the following summer of 2021. A single experimenter (D.J.C.) did all trials. Similar clothing was worn throughout. No trials were done in dim light, strong winds, or rain. A GoPro HERO7 Black camcorder was worn in a chest harness for all trials, and a smartphone was carried in one hand. Once a crow was located, the experimenter walked directly toward it at a steady walking pace (∼1.2 m/s) using 1 of 2 types of human gaze direction: a direct gaze at the focal crow, or an indirect gaze for which the experimenter's entire face was tilted down. During indirect gaze trials, video was streamed live from the GoPro to the smartphone to help the experimenter maintain a direct approach and to record when crows fled. The experimenter recorded the number of paces taken from the start of the approach until the focal crow fled and the total number of paces taken after reaching the original position of the focal crow. FID was the difference between these 2 measurements, while the latter is the starting distance. FID may vary with the attentional cost of monitoring an approaching person, which increases with the experimenter's starting distance and causes a longer FID (Blumstein 2003). Both FID and starting distance were converted to meters. We did not record alert distance, as only 1 experimenter was present during trials and video quality was insufficient to determine when crows became alert to the approach. The GPS location of the focal crow, date, flock size (conspecifics within 20 m), escape behavior (walking, hopping/running, or flying), and initial behavior of the focal crow (preening, scanning, and foraging) were also recorded.

#### Quantifying urbanization

Ecological studies often require different working definitions of “urban” depending on their goals (McIntyre et al. 2008). Our goals in quantifying urbanization were to facilitate comparison between studies of FID by choosing a single measure, and to quantify both the extent of crows' experiences with humans and the nature of those experiences (eg are crows tolerated or fed vs. chased or hunted) on their home territories, which they know well. We chose to use human population density as our measure of urbanization, as it is widely available and directly quantifies the number of people living in an area. We assumed that crows have more positive experiences with humans in areas where humans are more common and that the 2 are likely correlated. In support of this assumption, crow hunting is prohibited within city limits in New York but is legal and occurs between 1 September and 31 March in rural areas. We then determined population density (people/km^2^) around each trial site in QGIS (QGIS Development Team 2023) using data from the 2020 United States Census blocks (United States Census Bureau 2020) and the 2021 Canada Census dissemination areas (Statistics Canada 2021), the smallest geographic units for which population data are reported. Population density for a given trial was the sum of population densities for geographic units in the area, weighted by the proportion of the buffer they occupied. To determine the spatial scale at which experience with humans most strongly affects FID, we measured population density in a series of buffers with radii of 50 to 1,500 m in increments of 50 m.

#### Statistical analyses

We fit a series of linear models, each with Population Density at 1 buffer distance as a fixed effect. Each model also included as fixed-effects Flock Size, Date, the interaction of Population Density and Gaze, and Starting Distance. Previous work with American crows in Washington State found that starting distance did not affect FID (Clucas et al. 2013), but we chose to include it because starting distance affects FID in most bird species, including other corvids (Randler 2008; Novcić 2023), and our observations of crows in our area suggested that it was likely to have an effect. We centered and scaled continuous predictors. Initial behavior and escape type showed little variation (foraging in 136/145 trials and flying in 125/145 trials, respectively) and were excluded from the analysis. The *R*^2^ values from the resulting models were compared, and the model with the highest *R*^2^ value was retained. We checked for multicollinearity using the *car* package (Fox and Weisberg 2019). Predictors estimated with high certainty to have estimated effects near 0 were removed from the model.

## Results

### Object neophobia

We completed 3 baseline and 4 object trials with each of 10 urban and 10 rural families (*N* = 140 trials). Trials were completed for 8 families in 2019 and 12 families in 2020. The latency for the first crow in a family to come within 3 m of the bait/object was only measured in 2020.

The first crow in a family to approach within 3 m of the bait/object tended to arrive sooner in urban areas across all levels of Object (Figure S1). For example, in the absence of an object, the first crow in an urban family approached within 3 m of the bait/object at 4.23 times the rate of the first crow in a rural family (hazard ratio = 4.23). This indicates that the differences between urban and rural crow approach latencies could not be due solely to differences in neophobia and that our experimental design did not exclude nontarget behaviors (Réale et al. 2007; Greggor et al. 2015). Given rural crows' greater wariness of people, it is likely that the association of the experimental setup with the human experimenter made rural crows more reluctant than urban crows to approach. A similar pattern was observed for the latency for the first crow in a family to feed (hazard ratio = 1.81; Figure S2) and for the model-estimated probability of feeding (0.99 in urban crows and 0.97 in rural crows; Figure S3), although the magnitude of these differences was smaller. To distinguish responses to the objects from this baseline difference between urban and rural crows, Figs. 2–4 in the main text use model estimates for each object relative to the no-object condition within the same environment, with the no-object estimate set to 0 for Cox models and to 1 for the Bayesian model of feeding probability. The *P*-values for the Cox models and posterior probabilities for the Bayesian model correspond to the Environment × Object interactions. Figures S1–S3 show the uncentered model estimates, which retain the baseline urban–rural differences, along with *P*-values or posterior probabilities for the Environment contrast within each level of Object.

**Figure 2:**
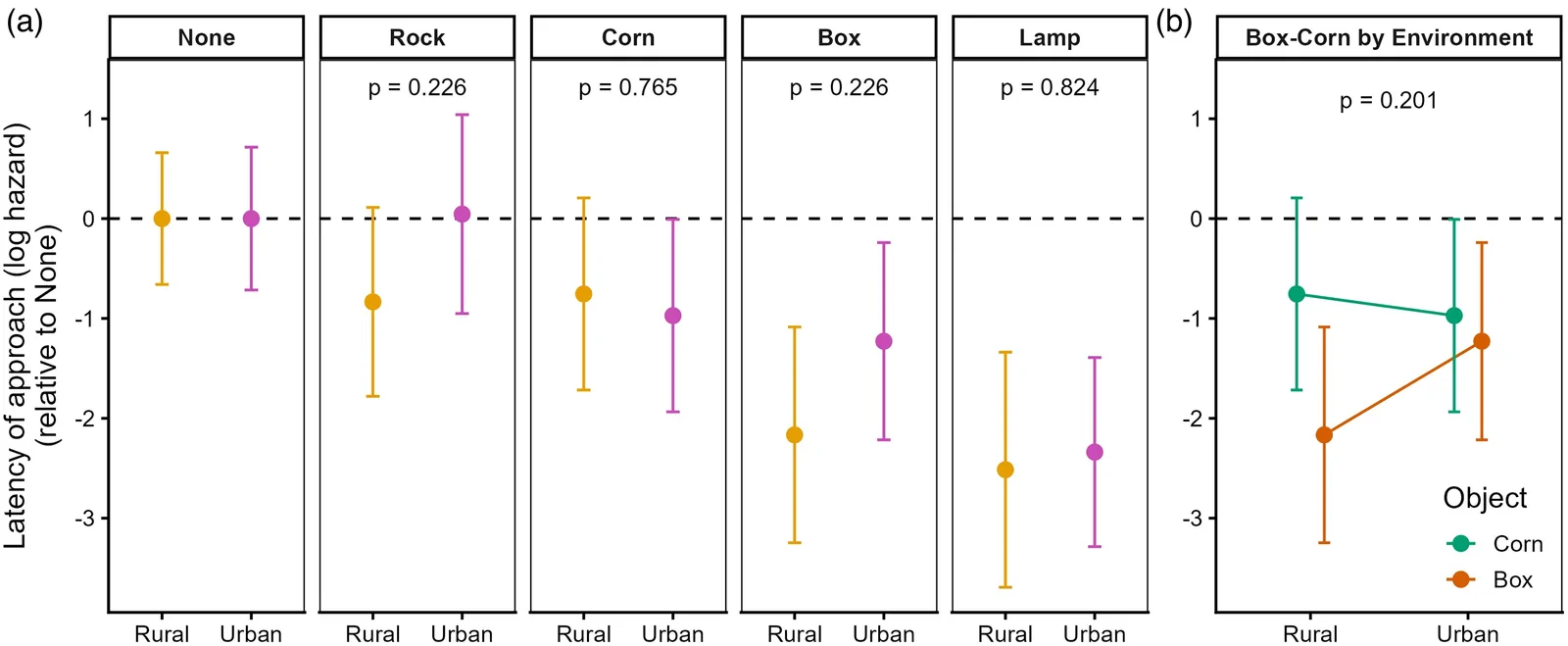
Estimated latency (log hazard) for the first individual in a family to approach within 3 m of bait/object (±95% CI) from a Cox proportional hazards mixed-effects model (*N* = 84, events = 81). Estimates are centered at 0 within each Environment when Object is none. a) Interaction of Environment and Object. Reference levels are rural and none. Objects are ordered according to their expected novelty in rural areas. b) The interaction between Environment and the Object levels box and corn, used to evaluate the hypothesis that different experiences in urban and rural areas result in the same objects having different levels of novelty.

**Figure 4:**
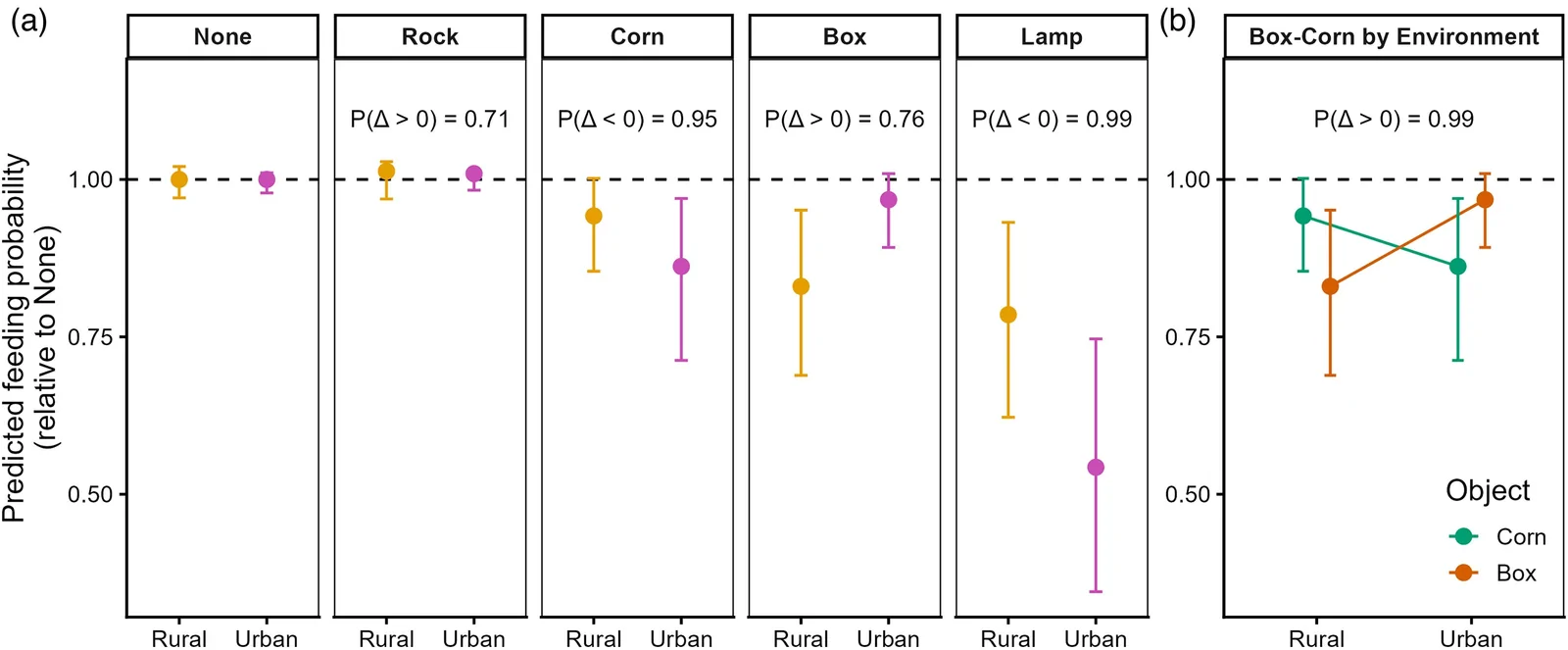
Predicted probabilities that an individual crow feeds after approaching within 3 m of the bait/object (±95% credible intervals) from a Bayesian binomial regression model (*N* = 140). Probabilities are centered at 1 within each Environment when Object is none. a) Interaction of Environment and Object. Reference levels are rural and none. Text labels indicate posterior probabilities for the direction of the interaction (Δ), where *P*(Δ < 0) indicates greater reductions in relative feeding probability for urban crows. Objects are ordered according to their expected novelty in rural areas. b) The interaction between Environment and the Object levels box and corn, used to evaluate the hypothesis that differing experiences in urban and rural areas result in these objects having different levels of novelty.

Neophobia did not differ between environments when measured by the latency to approach within 3 m of the lamp (Fig. 2a; Table 1; pairwise contrasts of Environment for each Object are in Table S2). There was limited evidence that the difference in approach latency between the box and corn varied between urban and rural crows. Although rural crows approached the corn more quickly than the box (Fig. 2b), urban crows had similar latencies for both objects and there was substantial uncertainty around all estimates. Estimated latencies tended to increase with the expected novelty of each object in each environment, but we cannot conclude that all adjacent pairs on the novelty scale differ, given the uncertainty around these estimates (pairwise contrasts of adjacent levels of Object within each Environment are in Table S3).

**Table 1:** Results of the Cox proportional hazards mixed-effects model for the latency for the first individual in a family to approach within 3 m of the bait/object.

Term	Estimate (β)	*e* ^β^	SE	*z*	*P*
Environment					
Urban	1.44	4.23	0.63	2.29	**0.022**
Object					
Rock	−0.83	0.43	0.50	−1.67	0.096
Corn	−0.76	0.47	0.51	−1.48	0.138
Box	−2.17	0.12	0.57	−3.81	**<0.001**
Lamp	−2.52	0.08	0.62	−4.03	**<0.001**
Environment × Object					
Urban: Rock	0.88	2.40	0.73	1.21	0.226
Urban: Corn	−0.22	0.81	0.73	−0.30	0.765
Urban: Box	0.94	2.55	0.78	1.21	0.226
Urban: Lamp	0.18	1.19	0.79	0.22	0.824

Estimates are on the log hazard scale. Positive values indicate a higher likelihood (shorter latency) of approach. Rural was the reference level for Environment, and none was the reference level for Object. *P*-values are in bold when *P* < 0.05. The exponentiated estimate (*e*^β^) is the hazard ratio. Standard deviation of the random intercepts for Family was 0.861.

The estimated latencies for the first crow in a family to feed after a family member approached within 3 m of the bait/object in lamp trials also suggest that neophobia levels were similar between urban and rural crows (Fig. 3a; Table 2 and Table S4). There was again only limited evidence that urban and rural crows responded differently to the corn and the box. The box produced greater latencies among rural crows, and the corn produced greater latencies among urban crows, but uncertainty was high relative to the magnitude of the differences (Fig. 3b). Within each environment, estimated latencies were again consistent with our expectations of the relative novelty of objects, but there was substantial uncertainty in comparisons of adjacent pairs on the novelty scale (Table S5).

**Figure 3:**
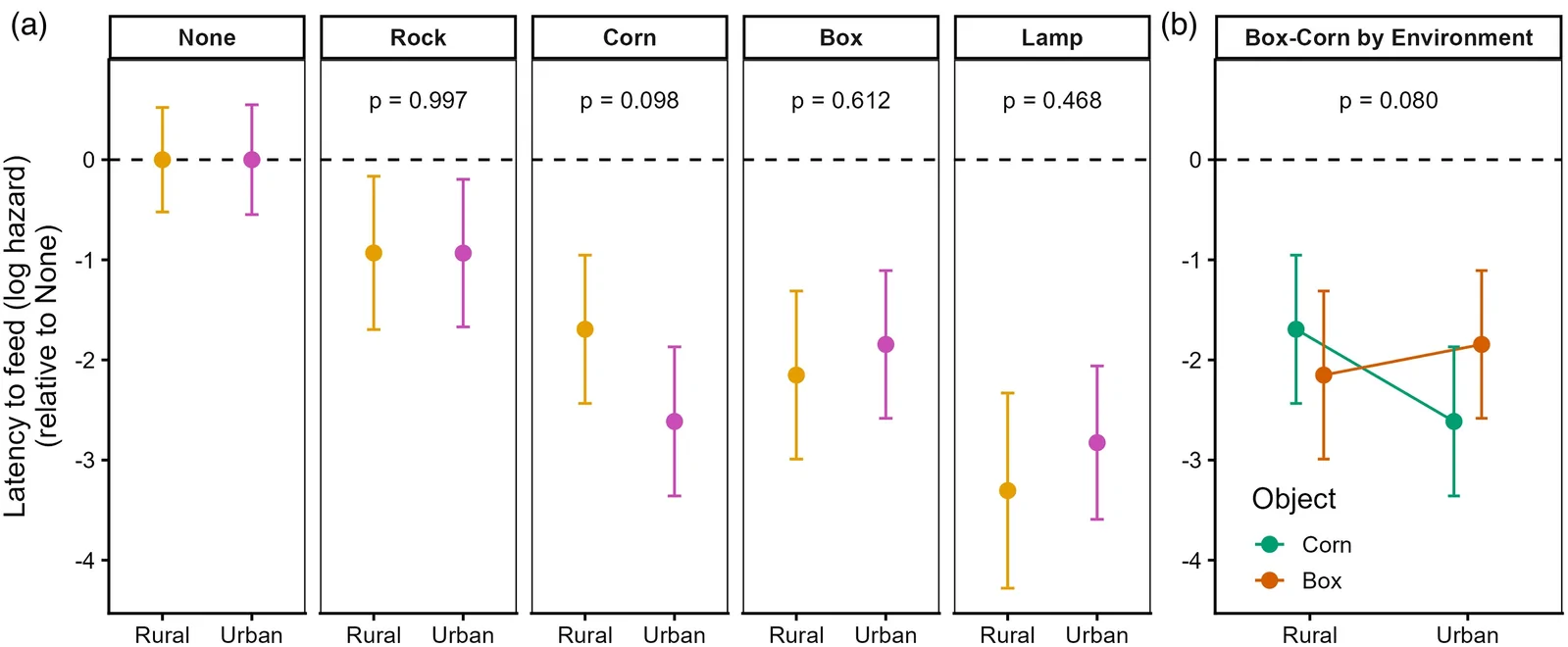
Estimated latency (log hazard) for the first crow in a family to feed after approaching within 3 m (±95% CI) from a Cox proportional hazards mixed-effects model (*N* = 140, events = 131). Estimates are centered at 0 within each Environment when Object is none. a) Interaction of Environment and Object. Reference levels are rural and none. Objects are ordered according to their expected novelty in rural areas. b) The interaction between Environment and the Object levels box and corn, used to evaluate the hypothesis that different experiences in urban and rural areas result in the same objects having different levels of novelty.

**Table 2:** Results of the Cox proportional hazards mixed-effects model for the latency for the first crow in a family to feed after approaching within 3 m of the bait/object.

Term	Estimate (β)	*e* ^β^	SE	*z*	*P*
Environment					
Urban	0.59	1.81	0.48	1.24	0.216
Object					
Rock	−0.93	0.39	0.41	−2.25	**0.025**
Corn	−1.69	0.18	0.40	−4.25	**<0.001**
Box	−2.15	0.12	0.46	−4.64	**<0.001**
Lamp	−3.31	0.04	0.54	−6.11	**<0.001**
Environment × Object					
Urban: Rock	−0.00	1.00	0.58	−0.00	0.997
Urban: Corn	−0.92	0.40	0.56	−1.65	0.098
Urban: Box	0.31	1.36	0.60	0.51	0.612
Urban: Lamp	0.48	1.61	0.66	0.73	0.468

Estimates are on the log hazard scale. Positive values indicate a higher likelihood (shorter latency) of feeding. Rural was the reference level for Environment and none was the reference level for Object. *P*-values are in bold when *P* < 0.05. The exponentiated estimate (e^β^) is the hazard ratio. The standard deviation of the random intercepts for Family was 0.851.

For the probability of feeding after coming within 3 m of the bait/object, the interaction between Object and Environment for the lamp indicated that urban crows were more neophobic than rural crows (Fig. 4a; Table 3), as urban crows reduced their feeding in lamp trials more than rural crows relative to trials with no object. Model-estimated feeding probability was slightly higher among urban crows (0.99) than rural crows (0.97) in trials with no object, but lower among urban crows (0.53) than rural crows (0.76) in lamp trials (Table S6). Urban and rural crows also responded differently to the box and the corn. The box caused greater reductions in feeding for rural crows, and the corn caused greater reductions in feeding for urban crows (Fig. 4b). Within each environment, estimated feeding probabilities tended to decline with increases in expected novelty, although uncertainty was again high in comparisons of adjacent objects along the novelty scale (Table S7).

**Table 3:** Results of a Bayesian binomial regression for the probability that a crow feeds after coming within 3 m of the bait/object.

Term	Estimate	Est. error	95% CrI
Intercept	3.56	0.49	**2.69, 4.59**
Environment			
Urban	0.98	0.81	−0.52, 2.67
Object			
Rock	0.74	1.10	−1.18, 3.17
Corn	−1.18	0.64	−2.42, 0.09
Box	−2.15	0.59	**−3.31, −1.01**
Lamp	−2.42	0.59	**−3.59, −1.27**
Environment × Object			
Urban: Rock	1.12	1.93	−2.35, 5.20
Urban: Corn	−1.59	0.99	−3.55, 0.33
Urban: Box	0.76	1.07	−1.35, 2.88
Urban: Lamp	−2.02	0.92	**−3.88, −0.30**

Estimates are on the log-odds scale. Positive values indicate higher odds of feeding. 95% CrI (credible intervals) that do not include 0 are bold. All parameters showed good convergence ($\hat{R}$ ≈ 1; bulk and tail ESS high). Rural was the reference category for Environment and none was the reference for Object. The standard deviation of the random intercepts for Family was 0.45 (95% CrI: 0.02 to 1.08).

Our litter survey in the 2 environments identified 341 human-made objects over the 3-wk survey period. The counts were similar in urban and rural areas at 169 and 172, respectively. The number of weeks each object was observed was higher in the rural survey (mean, 2.81 wks) than the urban survey (mean, 2.27 wks) (*t*-test: *t*_274.46_ = 7.039, *P* < 0.001) (Figure S4).

### Influences on FID

We conducted 145 FID trials. The model retained for analysis had a Population Density buffer radius of 800 m and an *R*^2^ of 0.314 (Figure S5). Starting Distance had a strong, positive effect on FID (Fig. 5; Table 4). There was no evidence for an effect of Date or Flock Size. There was a Population Density by Gaze interaction, but confidence intervals were wide, and both a small and a large effect were possible. To help interpret this interaction, we fit a reduced linear model without Date and Flock Size and with the dependent variables uncentered and unscaled (Table S8), then graphed the predicted interaction of Population Density and Gaze in Fig. 6. Estimates of FID for both a direct and indirect Gaze are high and similar at low levels of Population Density, but estimates diverge with increasing Population Density, as an indirect Gaze results in a steeper decline in FID relative to a direct Gaze, indicating that only crows in urban areas responded to human gaze.

**Figure 5:**
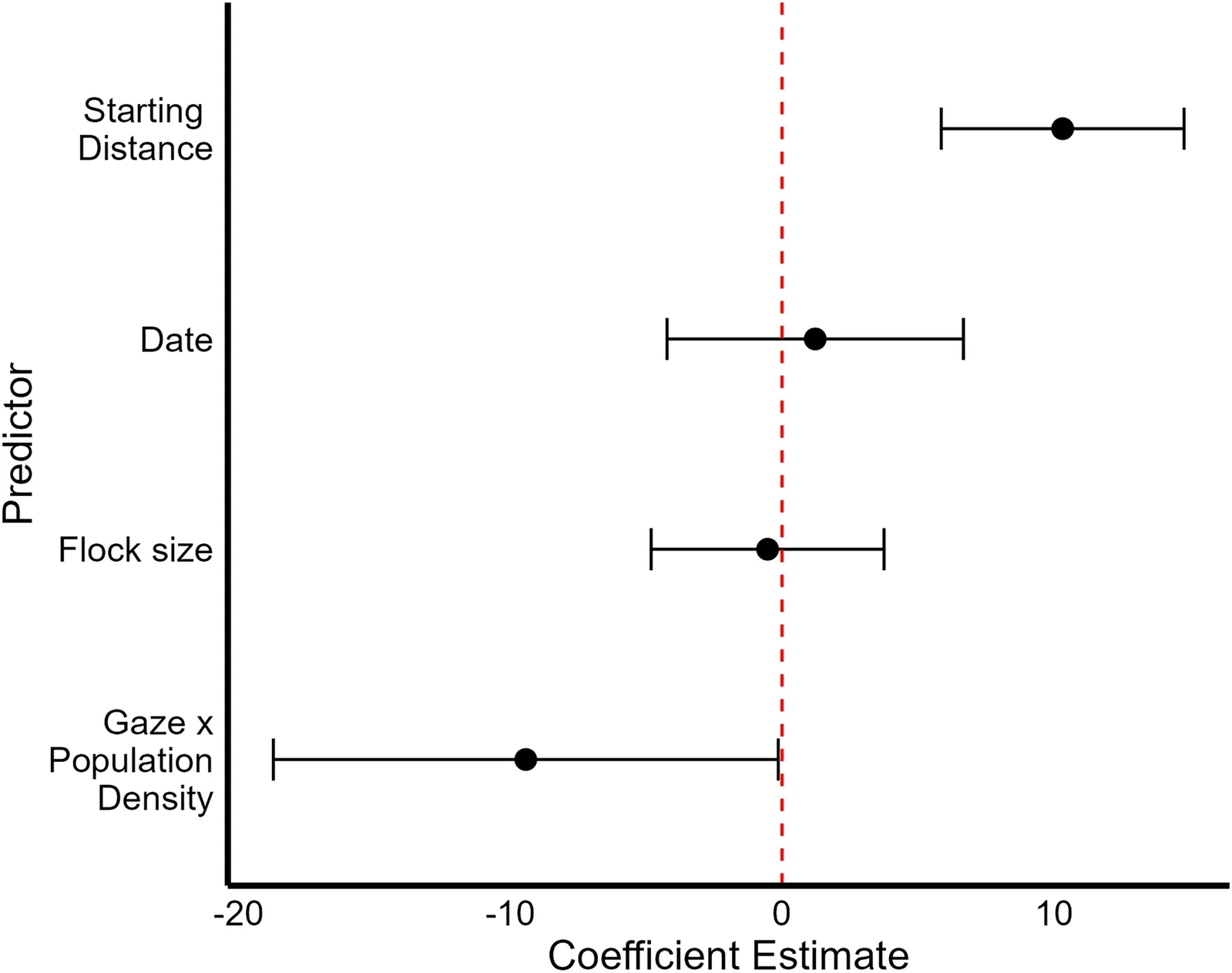
Selected coefficients with 95% confidence intervals from the full linear model of FID (*N* = 145). Continuous predictors are centered and scaled. Estimates represent the change in FID for an increase of 1 standard deviation of each predictor.

**Table 4:** Results of the linear model predicting FID with continuous predictors centered and scaled. Direct is the reference level for Gaze.

Terms	Estimate	SE	*t*	*P*
Intercept	44.60	3.28	13.59	**<0**.**001**
Population Density	−5.46	2.78	−1.96	0.052
Gaze				
Indirect	1.03	5.33	0.19	0.847
Starting Distance	10.33	2.26	4.57	**<0**.**001**
Flock Size	−0.53	2.17	−0.25	0.807
Date	1.22	2.76	0.44	0.658
Population Density × Gaze	−9.43	4.70	−2.01	**0**.**047**

*P*-values are in bold when *P* < 0.05.

**Figure 6:**
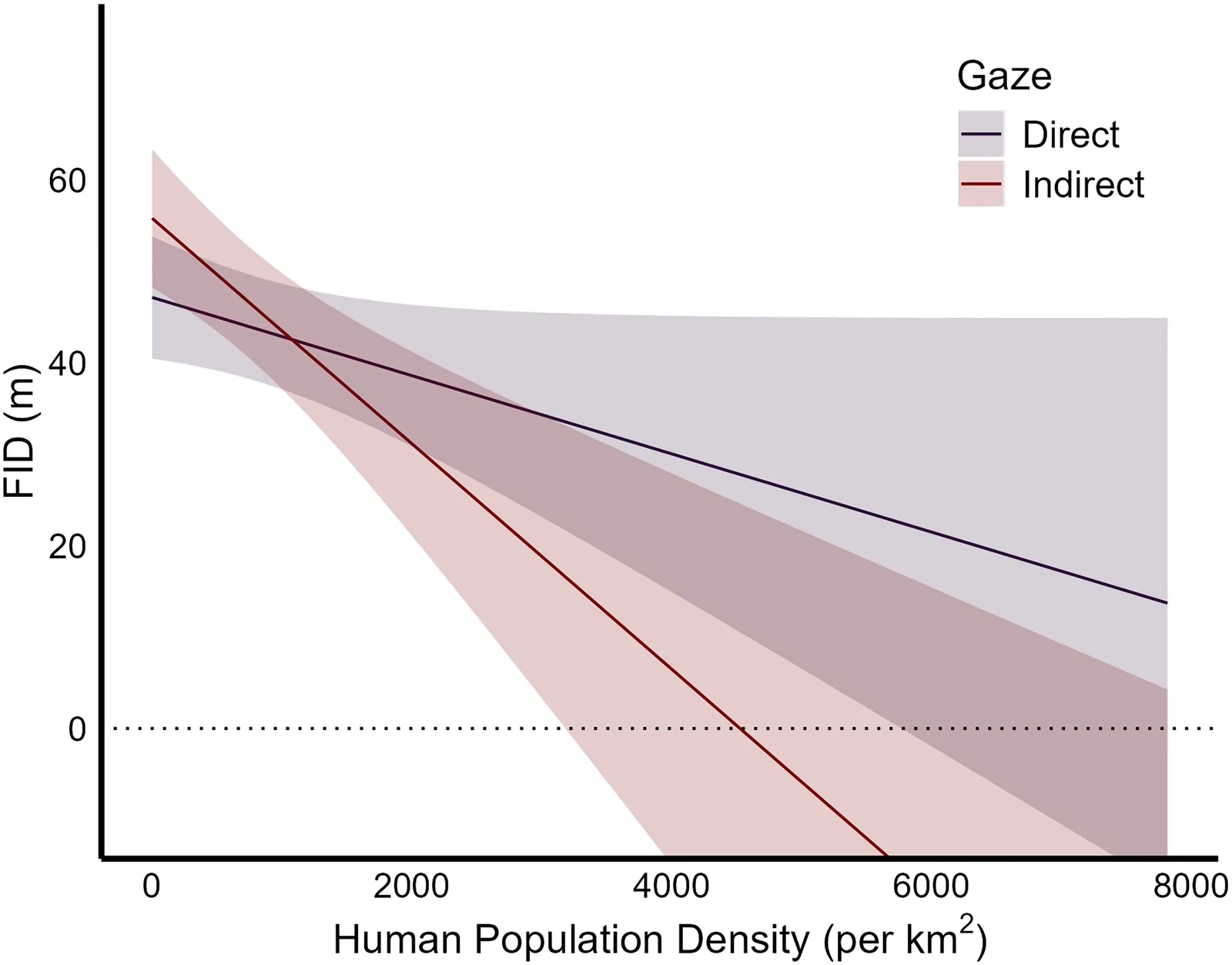
The model-predicted interaction of Gaze and Population Density (ie urbanization) from the reduced model of FID, using continuous predictors on their original scale (*N* = 145). Shaded areas are 95% confidence intervals.

## Discussion

### Object neophobia

The contrast in behavior of urban and rural crows varied with the object being presented and between measures of neophobia. Urban crows in this experiment appeared to be more neophobic than rural crows as determined by their responses to the lamp, but only for our group measure. The latency for the first crow in a family to approach within 3 m of the bait/object and the latency to feed, both of which reflect the response of the least neophobic crow in a family, suggested that urban and rural neophobia levels were similar. In contrast, the probability that family members fed, which measures neophobia levels among all crows that come within 3 m of the bait/object, indicated that neophobia levels were higher among urban crows. This suggests that there are crows with low neophobia levels in family groups in both urban and rural areas, but that neophobia is higher on average in urban areas.

Reduced urban neophobia levels were expected because neophobia may inhibit behavioral innovations (Webster and Lefebvre 2001; Sol et al. 2011; Cauchard et al. 2013), and innovation may be necessary for a species to invade a new environment (Sol et al. 2005). However, neophobia does not inhibit innovation in all species (Griffin and Guez 2014) and multiple factors can affect overall behavioral flexibility, including innovativeness, general learning ability, and the strength of neophilia relative to neophobia (Griffin et al. 2017). Corvids are highly exploratory (Heinrich 1995; Greenberg and Mettke-Hofmann 2001; Stöwe et al. 2006a, 2006b; Brown 2014), and American crows are excellent problem-solvers (Pendergraft et al. 2020; Townsend et al. 2022) and occasional tool-users (Caffrey 2000). Thus, high neophobia may not prevent crows from eventually taking advantage of novel resources but may function to slow initial approaches and protect against unknown dangers, consistent with the dangerous niche hypothesis (Greenberg and Mettke-Hofmann 2001; Mettke-Hofmann et al. 2013). Higher levels of neophobia among urban crows may therefore indicate that urban areas contain substantial unknown urban dangers, even for crows who know urban areas well.

It is also possible that neophobia is a barrier to innovation for American crows, that the individuals that initially colonized our urban study area had low levels of neophobia, and that higher levels of neophobia only became beneficial once the urban population was established. Several studies of birds have found behavioral differences between the individuals colonizing a new area and those in more established populations (Martin II and Fitzgerald 2005; Liebl and Martin 2014; Magory Cohen et al. 2020; Quesada et al. 2022). For example, common mynas (*Acridotheres tristis*) from an invasion front are more tolerant of novel foods than invasive mynas that are far from the front or in their native range (Magory Cohen et al. 2020). As American crows learn from conspecifics about both dangers (Cornell et al. 2012) and resources (Campbell-Smith 2015), young crows reared in established urban populations may learn from adults about what is useful and what is dangerous. For these individuals, the benefits of higher neophobia might outweigh any potential costs, such as decreased innovation (Griffin et al. 2017).

Beyond potential innovation-related benefits, expectations of reduced neophobia in urban populations are driven by the presence in urban areas of conditions that can reduce neophobia in captive animals (Corey 1978). For example, urban areas are often characterized as being spatially highly complex (Griffin et al. 2017) and frequently changeable over short timescales (Shochat et al. 2006; Alberti et al. 2017). Indeed, American crows in our urban study area seemingly experience high levels of spatial complexity; their territories often encompass several distinct habitats, such as stands of trees, parks, commercial areas, homes, and schools. Furthermore, our litter survey shows that turnover in small, artificial objects is higher in our more urban area. While the survey was limited to roadsides, it suggested that urban crows are exposed to constant turnover in the objects on their territories, many of which are presumably novel. Because we found no evidence that this has caused a reduction in neophobia, urban crows may be placing many of the objects they encounter into a single category rather than recognizing the unique properties of each.

As expected, we found that crows tend to respond with less aversion to objects that are more common in their environment. Rural crows were less averse to the corn and urban crows were less averse to the box across all measures, although the only measure that allows us to conclude this with confidence is the probability of feeding in a trial. Responses in proportion to the degree of novelty have been documented in captive animals such as lab rats (*Rattus norvegicus*), which consume less of a novel flavor as it becomes increasingly concentrated, independent of palatability (Strouthes and Navarick 1967; Navarick and Strouthes 1969; Domjan and Gillan 1976), and chimpanzees (*Pan troglodytes*), which spend more time handling objects with a greater number of novel characteristics (Menzel et al. 1961). To our knowledge, this is the first study to explicitly test whether responses to objects vary with their degree of novelty in a wild animal population. Confirming this relationship between experience, novelty, and aversion should draw attention to the difficulty of comparing neophobia levels in populations or individuals with different experiences.

We note that despite our intentions, we may not have accounted for differences in urban and rural experiences when choosing the lamp as our most novel object. We intended to choose an object without properties that are common to artificial objects and thus less novel to urban crows; however, the lamp is perfectly symmetrical around its vertical axis, and perfect symmetry is rare in natural objects. Our ability to measure differences in neophobia between urban and rural crows depends on comparisons in the lamp trials. If the lamp is more familiar to urban crows, neophobia levels among urban crows may be even higher relative to rural crows than our results indicate.

Properly accounting for experience when choosing a novel object will also make comparisons between studies of neophobia easier. For example, Merz et al. (2025) found no differences along an urban–rural gradient in the latency of the first American crow in a group to contact bait placed next to a GoPro HERO4 Black video camera. This latency is equivalent to the sum of our 2 latencies (ie to approach and to feed) and was generally shorter than the summed latency we observed in lamp trials. Comparisons between studies are difficult because the GoPro was not necessarily equally novel to all crows along the urban–rural gradient. Merz et al. (2025) collected data outside of the breeding season when groups may contain a mix of individuals from urban and rural areas, and of locals and migrants (Townsend et al. 2018), all of whom likely have different levels of experience with artificial objects and their properties. As a result, it is also unclear how novel the GoPro was relative to the lamp used here. Therefore, it is difficult to say if the lack of urban–rural differences in Merz et al. (2025) and the shorter latencies for the GoPro than for the lamp are the result of different levels of experience with artificial objects, of differences in neophobia levels, or of other methodological differences, including object size, social context, or testing season (Miller et al. 2015; Greggor et al. 2016a, 2016b). One other study has compared urban and rural corvid neophobia (Greggor et al. 2016a, ). As in Merz et al. (2025), the novel object in that study was made of artificial materials which may be more familiar to urban individuals.

### Influences on FID

Consistent with a previous study (Clucas and Marzluff 2012), we found urban crows allow much closer approaches than rural crows, and that urban crows use human gaze to adjust their FIDs, while rural crows do not. While there was substantial uncertainty, in moderately urban areas (∼5,000 people per square km) our model predicts that an indirect gaze would result in an FID near 0, while a direct gaze is predicted to result in an FID around 25 m. The use of gaze by American crows is consistent with studies of hand-raised corvids, including jackdaws (*Coloeus monedula)* (von Bayern and Emery 2009), rooks (Schmidt et al. 2011), and ravens (Bugnyar et al. 2004; Zeiträg and Osvath 2023), which demonstrate that corvids can respond to a human's face and eye orientation. Similar differential responses to direct and indirect gazes during FID trials have been found in wild populations of other bird species, such as hadeda ibises (*Bostrychia hagedash*). In a study in South Africa, all ibises reacted to a direct gaze from the experimenter by moving away from their approach. In contrast, only 20% of ibises moved away from the experimenter's approach during indirect gaze trials (Bateman and Fleming 2011). To our knowledge, the present study is the first to demonstrate population differences in the use of human gaze to determine FID. The difference in FID for direct and indirect gaze trials in this study was larger than that from a prior study of urban crows in Seattle, which found that FID was on average 3.2 m less for approaches with an indirect gaze at an alert distance of 12.30 m (Clucas et al. 2013). Other population differences are also apparent. Clucas et al. (2013) reported that crows in Seattle typically allowed approaches within 10 m, while we recorded only 8 approaches under 10 m in 145 trials (unadjusted FID mean ± SE = 45.3 ± 4.5 m). It is unclear what drives these differences, but Seattle crows have lower body mass (Verbeek et al. 2024), which may result in quicker takeoffs (Dial et al. 2008), and the larger urban area and higher human population density in parts of the Seattle study area (up to 12,900 people per square km) may have resulted in more habituation to human approaches.

Human population density measured within 800 m of an FID trial explained the most variance in FID. This is somewhat surprising. For most American crows, 800 m likely encompasses their territory, which they know well, as well as parts of neighboring territories, which they are likely familiar with but rarely use during the breeding season. Furthermore, birds can use fine-scale experiences to determine their escape behavior. For instance, common blackbirds (*Turdus merula*) tested in parks will adjust their FID according to the time of day, with closer approaches tolerated during times when pedestrians are typically more common (Rodriguez-Prieto et al. 2009). Such fine-scale decision-making should allow birds to minimize the costs of fleeing while avoiding unnecessary risks. Thus, 800 m seems broad considering the spatial scale on which crows experience people and the economics of decision-making. However, this finding may indicate that variation in human presence over short distances does not reflect variation in the risk posed by humans. For instance, a person walking in an isolated vacant city lot that is rarely trafficked and has a population density near 0 may pose no greater risk than a person walking 1 block away on a busy residential street where population density is high. If so, crows may make FID decisions that best balance flight costs and predation risk by averaging their experiences over a larger area. Further decisions about the risk from an approaching person can then be made based on that person's behavior (Clucas et al. 2013; this study) and appearance (Marzluff et al. 2010). Alternatively, human population density itself may fail to quantify human presence at small spatial scales because it measures how many people reside in an area rather than how many people are present there. For example, urban parks or shopping centers can have population densities near 0 even though pedestrians are common and unthreatening. Population density measured on a small scale around an FID trial in such areas would therefore fail to reflect human presence. In contrast, if population density were measured on a larger scale, some of this inaccuracy might be averaged out if the larger area included adjacent urban areas, such as residential neighborhoods, where population density more accurately reflects human presence. Thus, human population density measured on a larger scale could better predict crow FIDs even if crows are using their experiences with humans at smaller scales to make FID decisions.

## Conclusions

We found evidence that urban American crows have higher neophobia levels than rural crows, although whether or how higher neophobia helps crows in urban environments remains unclear. Determining whether neophobia inhibits innovation, or whether there are greater numbers of novel threats in urban environments, would help resolve this question. We also found that both urban and rural crows showed increasing aversion to increasingly novel objects when novelty was determined by how common an object was in each environment. This result should draw attention to the need to account for population differences in experience when selecting novel objects. Valid comparisons of urban and rural neophobia levels should begin by identifying what types of objects are present in each environment and which object properties are associated with artificial objects. In our FID experiment, the use of human gaze increased with urbanization, while crows in very rural areas fled at great distances regardless of gaze direction. Last, crows used their experiences with people in a relatively large area to inform FID decisions. Understanding how well experiences within a small area can inform crows about the risk posed by a typical person is key to interpreting this result.

## Supplementary Material

arag135_Supplementary_Data

## Data Availability

Analyses reported in this article can be reproduced using the data provided by Colucci and Clark (2026).
