# Supplementary material for: Experience affects novel object aversion and the use of human cues in rural and urban American crows
Source: Behav Ecol. 2026 Oct 22;37(6):arag135. doi: 10.1093/beheco/arag135 (PMC13755870; doi:10.1093/beheco/arag135)
Supplement: Supplementary file 1 [file arag135_supplementary_data.pdf]

Supplemental Material

Table S1. Human population density and urban land cover within 245.5 m of each experimental neophobia site.

Environment	Family ID	People / km^2	% urban land cover
Rural	BLUE	25.62	16.67
Rural	CADY	38.90	12.37
Rural	CURV	29.33	8.25
Rural	DRYL	78.19	13.86
Rural	FARK	63.69	12.24
Rural	MATT	17.67	9.09
Rural	MEEK	48.57	14.00
Rural	THOM	17.53	8.00
Rural	TRAK	14.69	6.12
Rural	WHIL	48.32	14.29
Urban	BIRC	615.45	53.06
Urban	BRAN	1094.41	94.00
Urban	BURL	1476.18	100.00
Urban	DONE	400.58	77.55
Urban	HORC	687.64	98.98
Urban	POST	149.24	96.94
Urban	ROTA	557.40	71.00
Urban	SALE	396.06	44.90
Urban	SWIM	13.54	44.90
Urban	THOR	19.87	96.00

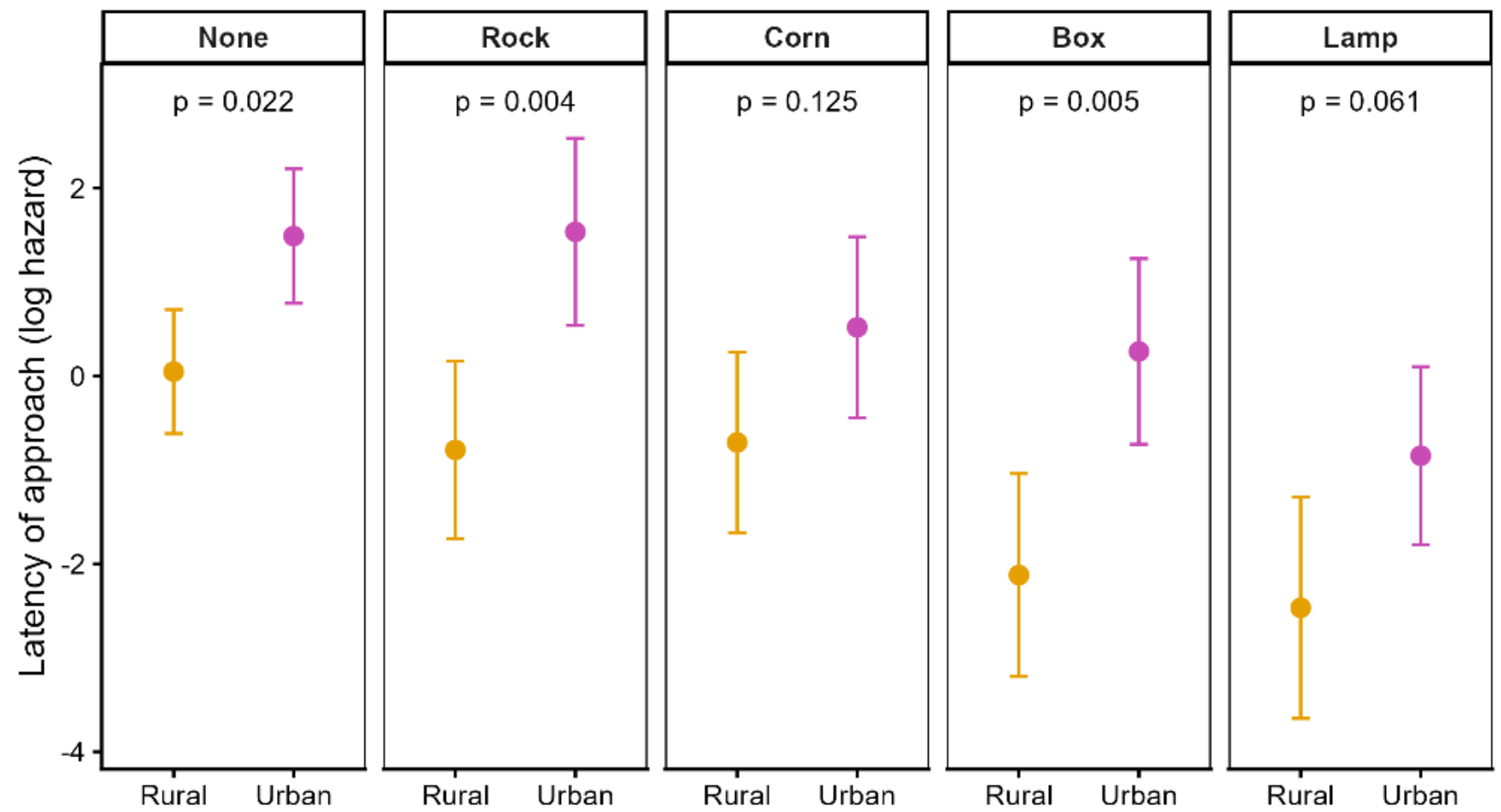

Figure S1. Estimated latency (log hazard) for the first individual in a family to approach within 3 m of bait/object (± 95% CI) from a Cox proportional hazards mixed-effects model (N = 84, events = 81). Comparison of urban and rural families by Object. Objects are ordered according to their expected novelty in rural areas.

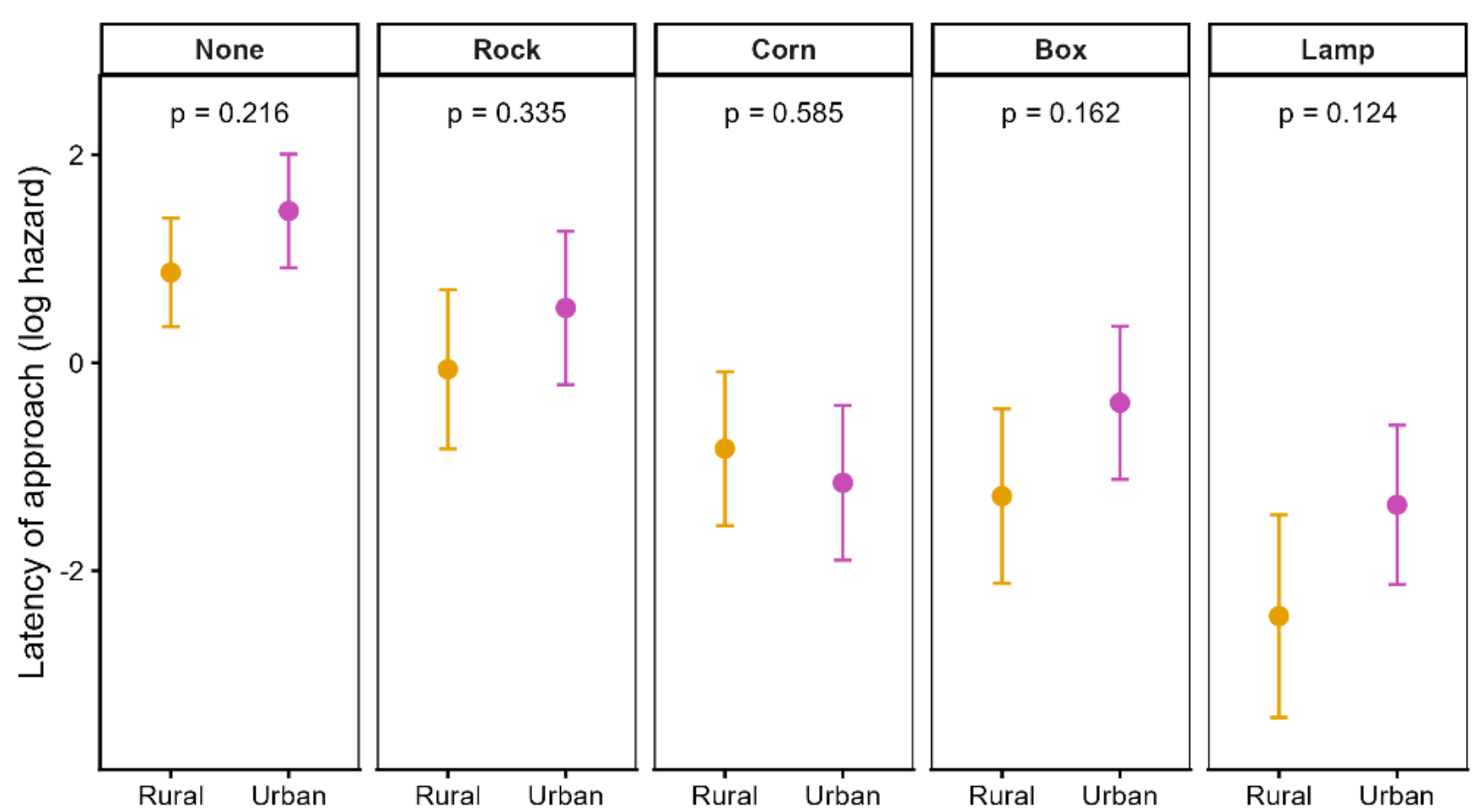

Figure S2. Estimated latency (log hazard) for the first crow in a family to feed after approaching within 3 m (± 95% CI) from a Cox proportional hazards mixed effects model (N = 140, events = 131). Comparison of urban and rural families by Object. Objects are ordered according to their expected novelty in rural areas.

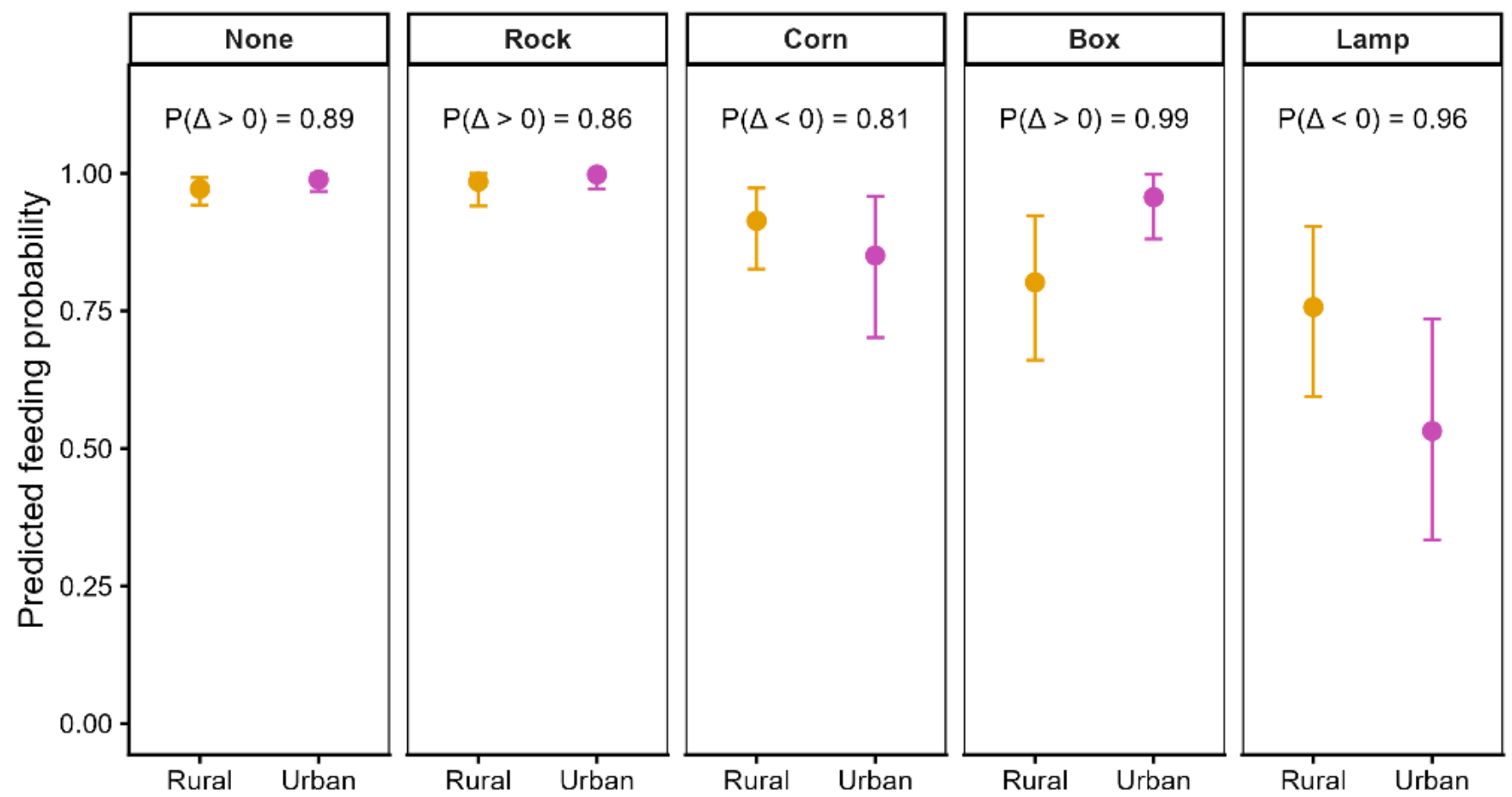

Figure S3. Predicted probabilities that an individual crow feeds after approaching within 3 m of the bait/object (± 95% credible intervals) from a Bayesian binomial regression model (N = 140). Comparison of urban and rural families by Object. Objects are ordered according to their expected novelty in rural areas. Text labels indicate posterior probabilities for the direction of the difference (Δ), where P(Δ<0) indicates lower feeding probability for urban crows.

Table S2. Pairwise contrasts of environments for each object from the Cox proportional hazards mixed-effects model of the latency for a family to approach within 3 m of the bait/object. Estimates are on the log hazard scale. Negative values indicate a lower likelihood (longer latency) of approach for rural relative to urban families. Holm adjustments. P-values are in bold when $P < 0.05$. The exponentiated estimate (e^{β}) is the hazard ratio.

Object	Estimate (β)	e^{β}	*SE*	z	*p*
None	-1.44	0.24	0.63	-2.29	**0.022**
Rock	-2.32	0.10	0.81	-2.86	**0.004**
Corn	-1.23	0.29	0.80	-1.53	0.125
Box	-2.38	0.09	0.85	-2.80	**0.005**
Lamp	-1.62	0.20	0.86	-1.87	0.061

Table S3. Planned pairwise contrasts testing hypothesized differences among objects within each environment from the Cox proportional hazards mixed-effects model of the latency for a family to approach within 3 m of the bait/object. Estimates are on the log hazard scale. Positive values indicate a greater likelihood (shorter latency) of approach for the first object relative to the second. P-values are in bold when $P < 0.05$. The exponentiated estimate (e^{β}) is the hazard ratio.

Contrast	Environment	Estimate (β)	e^{β}	*SE*	z	*p*
None - Rock	Rural	0.83	2.30	0.50	1.67	0.096
Rock - Corn	Rural	-0.08	0.92	0.61	-0.13	0.896
Corn - Box	Rural	1.41	4.10	0.66	2.14	**0.033**
Box - Lamp	Rural	0.35	1.42	0.69	0.50	0.614
None - Rock	Urban	-0.04	0.96	0.52	-0.09	0.931
Rock - Box	Urban	1.27	3.57	0.64	2.00	**0.046**
Box - Corn	Urban	-0.26	0.77	0.63	-0.41	0.682
Corn - Lamp	Urban	1.37	3.92	0.61	2.24	**0.025**

Table S4. Pairwise contrasts between environments for each object from the Cox proportional hazards mixed-effects model of the latency for the first crow in a family to feed after approaching within 3 m of the bait/object. Estimates are on the log hazard scale. Negative values indicate a lower likelihood (longer latency) of approach for rural relative to urban families. Holm adjustments. P-values are in bold when $P<0.05$. The exponentiated estimate (e^{β}) is the hazard ratio.

Object	Estimate (β)	e^{β}	*SE*	z	*p*
None	-0.59	0.55	0.48	-1.24	0.216
Box	-0.90	0.41	0.64	-1.40	0.162
Corn	0.33	1.39	0.60	0.55	0.585
Lamp	-1.07	0.34	0.70	-1.54	0.124
Rock	-0.59	0.55	0.61	-0.96	0.335

Table S5. Planned pairwise contrasts testing hypothesized differences among objects within each environment from the Cox proportional hazards mixed-effects model of the latency for the first crow in a family to feed after approaching within 3 m. Estimates are on the log hazard scale. Positive values indicate a greater likelihood (shorter latency) to feed for the first object relative to the second. P-values are in bold when $P < 0.05$. The exponentiated estimate (e^{β}) is the hazard ratio.

Contrast	Environment	Estimate (β)	e^{β}	*SE*	z	*p*
None - Rock	Rural	0.93	2.54	0.41	2.25	**0.025**
Rock - Corn	Rural	0.76	2.14	0.49	1.55	0.122
Corn - Box	Rural	0.46	1.58	0.52	0.88	0.378
Box - Lamp	Rural	1.15	3.17	0.60	1.92	0.055
None - Rock	Urban	0.93	2.54	0.41	2.28	**0.023**
Rock - Box	Urban	0.91	2.49	0.47	1.95	0.051
Box - Corn	Urban	0.77	2.16	0.47	1.65	0.099
Corn - Lamp	Urban	0.21	1.24	0.47	0.45	0.654

Table S6. Odds ratios with 95% CrI (credible intervals) comparing environments for each object from the Bayesian binomial regression for the proportion of crows that feed after coming within 3 m of the bait/object. Odds ratios >1 indicate lower odds of feeding in urban relative to rural environments. 95% CrI that do not include 1 are in bold.

Object	Odds ratio	95% CrI
None	0.39	0.02, 1.34
Rock	0.14	<0.01, 2.38
Corn	1.86	0.21, 6.08
Box	0.18	**<0.01, 0.67**
Lamp	2.76	0.47, 7.95

Table S7. Odds ratios with 95% CrI (credible intervals) comparing objects within each environment from the Bayesian binomial regression for the proportion of crows that feed after coming within 3 m of the bait/object. Odds ratios >1 indicate higher odds of feeding for the first object relative to the second. 95% CrI that do not include 1 are in bold.

Contrast	Environment	Odds ratio	95% CrI
None - Rock	Rural	0.53	<0.01, 2.48
Rock - Corn	Rural	6.09	0.29, 49.53
Corn - Box	Rural	2.59	0.47, 7.30
Box - Lamp	Rural	1.31	0.25, 3.37
None - Rock	Urban	0.19	<0.01, 2.91
Rock - Box	Urban	20.96	0.04, 1,086.42
Box - Corn	Urban	3.90	0.24, 19.00
Corn - Lamp	Urban	5.05	0.65, 15.62

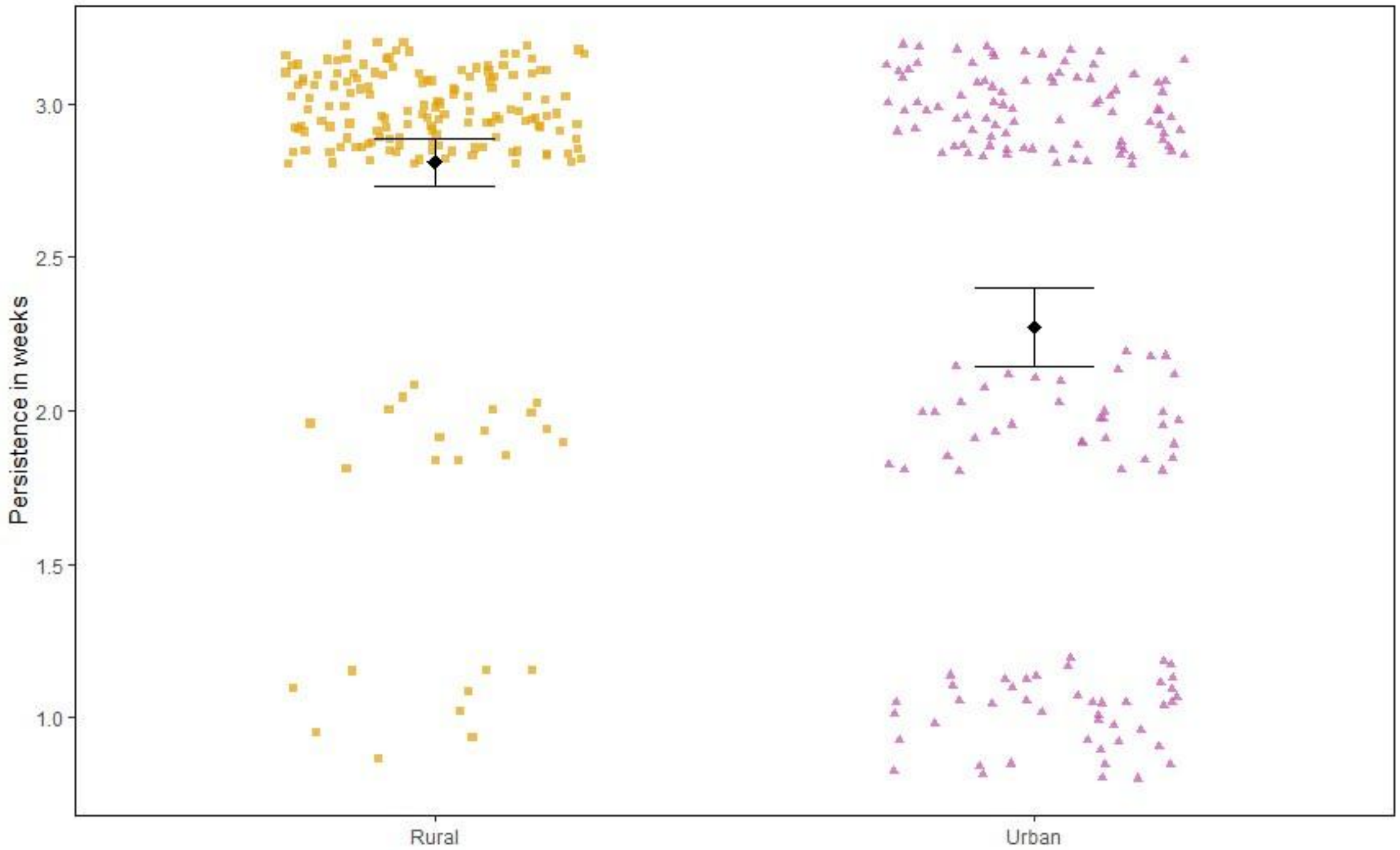

Figure S4. The persistence of each human-made object counted over a three-week survey along urban and rural roadsides. 95% confidence intervals. T test: $t_{274.46} = 7.039$, $P < 0.001$. N = 341.

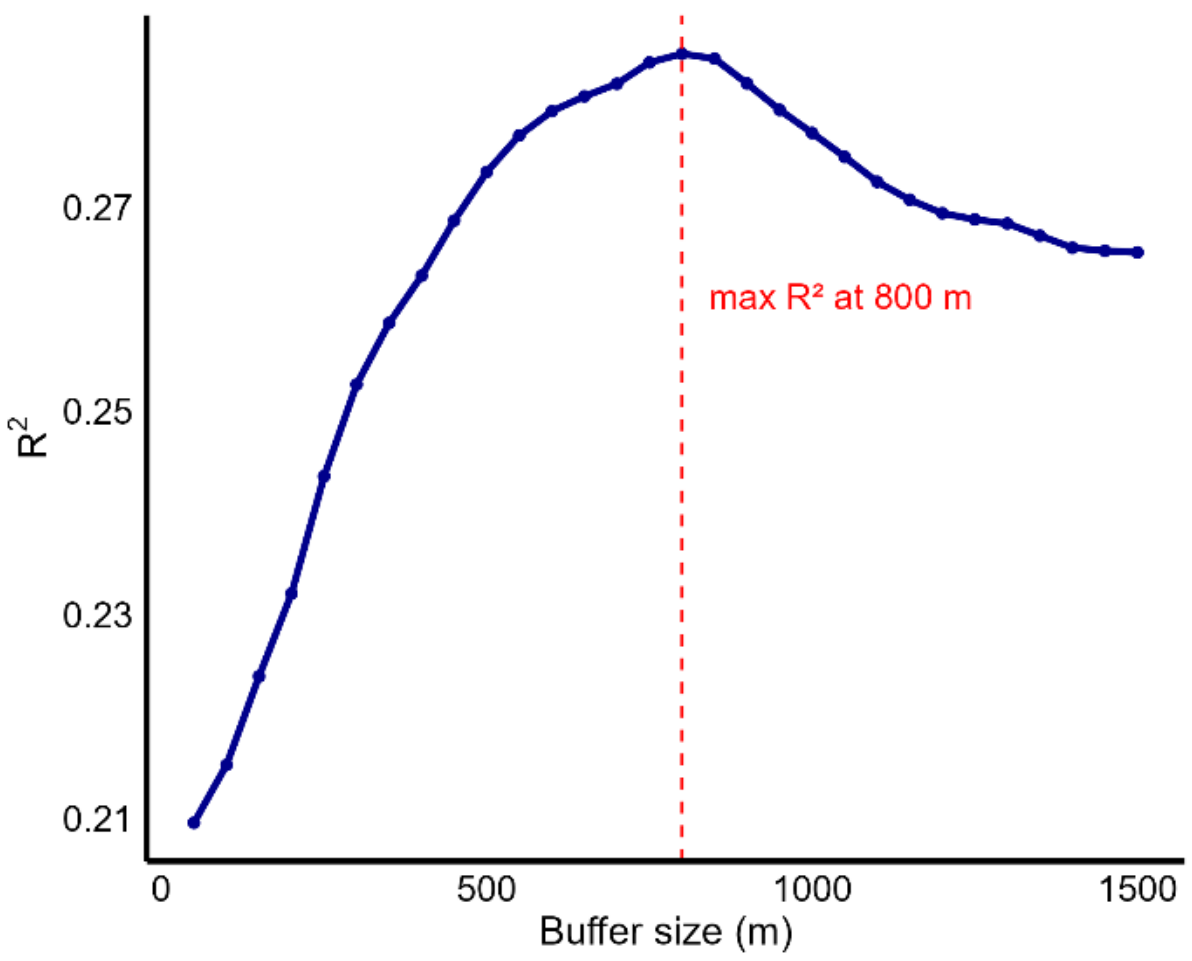

Figure S5. Graph of R^2 values from the series of linear models predicting FID, each with Population Density measured at one buffer distance around FID trials.

Table S8. Reduced linear model of FID with continuous predictors on their original scale. Direct is the reference level for Gaze. *P*-values are in bold when $P < 0.05$.

Term	Estimate	*SE*	*t*	*p*
Intercept	43.90	2.89	15.20	**<0.001**
Population Density	-5.14	2.66	-1.93	0.055
Gaze				
Indirect	2.52	4.22	0.60	0.552
Starting Distance	10.18	2.22	4.57	**<0.001**
Population Density x Gaze	-9.67	4.53	-2.13	**0.035**
